# Bartonella Endocarditis in a Pediatric Patient With Aortic Valvular Disease and Embolic Stroke: A Case Report and Review of the Literature

**DOI:** 10.7759/cureus.98965

**Published:** 2025-12-11

**Authors:** Omoadoni D Emeagui, Gitanjali Rebello, Rafay A Afzal, Kendria C Hall, Rebecca E Pratt, Karl O. A Yu

**Affiliations:** 1 Pediatrics, University at Buffalo Jacobs School of Medicine and Biomedical Sciences, Buffalo, USA; 2 Pediatric Infectious Diseases, University at Buffalo Jacobs School of Medicine and Biomedical Sciences, Buffalo, USA; 3 Biomedical Informatics, University at Buffalo Jacobs School of Medicine and Biomedical Sciences, Buffalo, USA; 4 Pediatric Cardiology, University at Buffalo Jacobs School of Medicine and Biomedical Sciences, Buffalo, USA; 5 Pediatric Infectious Diseases, SUNY at Buffalo Jacobs School of Medicine, Buffalo, USA

**Keywords:** aortic valvular disease, bartonella endocarditis, embolic stroke, microbial cell-free dna testing, pediatric

## Abstract

Culture-negative endocarditis leading to embolic stroke is a rare clinical etiology in pediatrics. This is a case of a 12-year-old male with a history of bicuspid aortic valve and mild asymptomatic aortic stenosis who presented with flu-like symptoms, right-sided hemiplegia, and aphasia. Although he initially presented with symptoms of arterial ischemic stroke, he was found to have infective endocarditis (IE) secondary to *Bartonella henselae*, confirmed by echocardiogram and plasma microbial cell-free DNA (mcf-DNA) testing. Initial antimicrobial treatment per standard guidelines was unsuccessful. Hence, second-line antimicrobials were commenced, to which he responded appropriately. His clinical course markedly improved, with recovery of neurological function within three weeks. He continued treatment of endocarditis and bartonellosis in an outpatient setting with significant clinical response until eventual cardiac surgery for valve replacement. Although the diagnosis and management of blood culture-negative IE can be very challenging and associated with morbidity, mcf-DNA testing has been shown to be helpful in establishing an accurate diagnosis and promoting appropriate antimicrobial management.

## Introduction

Infective endocarditis (IE) is an infection involving the endocardial lining of the heart, most commonly affecting the heart valves (native or prosthetic) or implanted cardiac devices, caused by pathogenic microorganisms of bacterial, viral, or fungal origin [1,2]. IE occurs less frequently in children than in adults but is associated with significant morbidity and mortality due to its often atypical presentation, prolonged course of treatment, and potential for severe complications [2]. The clinical picture of IE may be insidious, with fever, night sweats, a new cardiac murmur, and weight loss often being the only presenting symptoms [2]. Several hallmark signs, such as Janeway lesions, Osler nodes, splinter hemorrhages, and Roth spots, may also be present [2,3]. The modified Duke Criteria, which comprise clinical, imaging, and bacteriological findings, are the standard for diagnosis [4,5]. IE usually occurs in two forms: acute IE, which develops suddenly and may become life-threatening within days, and subacute or chronic IE, which develops slowly over weeks to several months [4,5].

The primary approach to diagnosing IE relies on culture-based methods. However, 20% of cases are blood culture-negative endocarditis (BCNIE), presenting a major diagnostic challenge in clinical practice and resulting in delayed diagnosis with increased risk of morbidity and mortality [4-6]. Prior antibiotic use accounts for 35-74% of the prevalence of BCNIE [7]. Therefore, recent advances have led to the development of non-culture-based diagnostics capable of identifying specific underlying microbes in blood culture-negative cases, thereby refining treatment regimens [7].

Serological assays such as indirect fluorescent antibody tests, enzyme-linked immunosorbent assays, and complement fixation tests have proven valuable in detecting fastidious organisms or pathogens that fail to grow in standard culture conditions [7,8]. Recent reports indicate that indirect immunofluorescence assays successfully identified organisms such as *Coxiella burnetii *[8].

Advanced molecular diagnostic methods have significantly improved the detection of IE [9]. These include organism-specific PCR assays, broad-range PCR targeting conserved regions such as the bacterial 16S rRNA gene, targeted metagenomic sequencing, and shotgun metagenomics [6,7,9]. The Karius test, developed by Karius, Inc. (Redwood City, CA, USA), detects microbial cell-free DNA (mcf-DNA) by next-generation sequencing from plasma. Recent studies suggest that mcf-DNA testing reliably identifies causative pathogens in cases of culture-negative IE, making the Karius test a valuable diagnostic tool in BCNIE [7,9].

*Bartonella *is a commonly reported cause of BCNIE. *Bartonella henselae *is often transmitted to humans by cats or dogs through scratches contaminated with feces from *B. henselae*-infected fleas [2]. When treating *B. henselae *infections, the IDSA recommends doxycycline or erythromycin monotherapy for bacteremia or osteomyelitis and dual therapy with doxycycline and rifampin for CNS infections or confirmed *Bartonella *endocarditis, with a total duration of therapy extending up to three months [10].

IE occurs less frequently in children than in adults [2]. In pediatric cases, the primary predisposing factor is congenital heart disease (CHD), while other notable risk factors include rheumatic heart disease (RHD) and bacteremia resulting from hospital-acquired infections [2]. However, with the increased use of invasive medical procedures and prolonged central venous catheter placement, the incidence of IE among individuals without pre-existing heart disease has been rising [10,11].

Among the complications of IE, stroke remains one of the most concerning, usually resulting from occlusion of intracerebral vessels following embolization of endocardial vegetations [12,13]. Here, we present a case of a child with a history of bicuspid aortic valve with aortic stenosis (no prior interventions) who developed and was treated for systemic *B. henselae *infection with endocarditis complicated by cerebrovascular disease.

## Case presentation

An early adolescent boy with a past medical history of bicuspid aortic valve and aortic stenosis presented with a history of fever, cough, and cold two weeks prior, followed by features of an acute embolic stroke. He was referred from an outlying emergency department. He had recovered from his upper respiratory tract symptoms when he developed confusion, mild drooping of the right side of his mouth, and numbness in his right upper arm and leg. His initial brain CT scan was normal, but his symptoms progressed, prompting referral to a tertiary care children’s hospital for further management.

A repeat CT with contrast showed large acute ischemic changes in the left middle cerebral artery territory. He was admitted for supportive management, as he was outside the thrombolytic window. Although he had been fever-free for 10 days prior to presentation, he was febrile at admission. History was notable for residing in a Lyme-endemic area, but he had no recent travel, camping history, or exposure to cats or dogs. There was no significant family history.

On examination, he was tachycardic, normotensive, and febrile, with petechiae and Janeway lesions on the bilateral palms and soles. Cardiac auscultation revealed a systolic ejection murmur at the right upper sternal border. Neurologic examination demonstrated verbal apraxia, right hemiparesis, right upper motor neuron facial nerve palsy, and right hypoglossal nerve palsy, with the tongue deviating to the right.

Investigations

**Figure 1:**
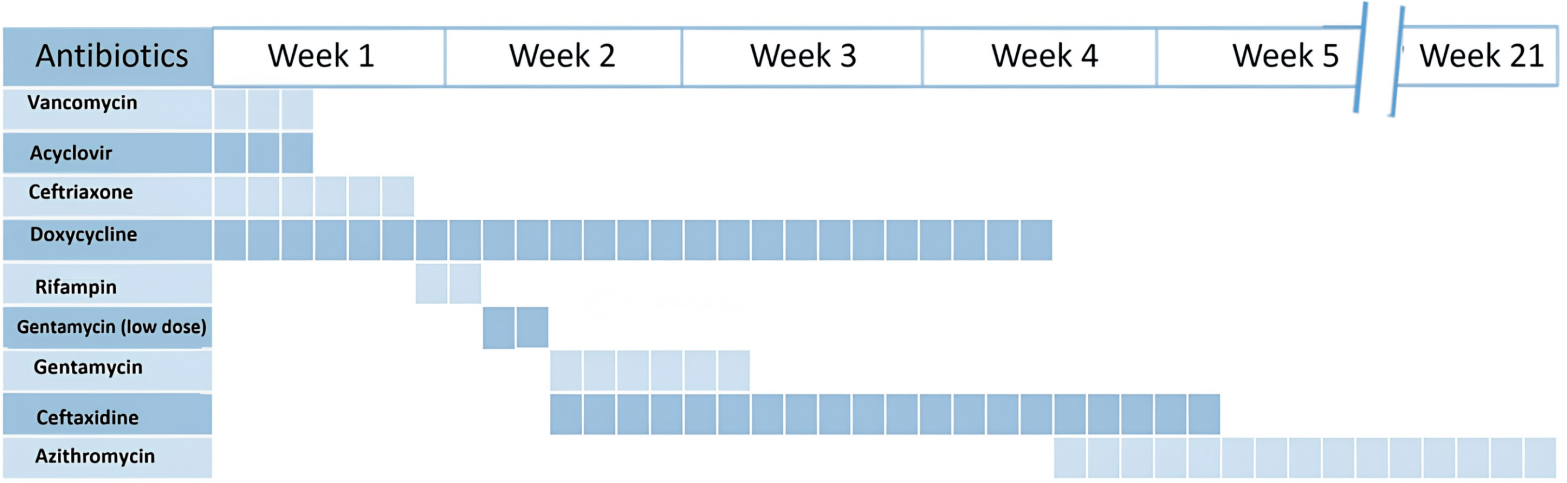
Chronology of the antibiotic regimen

**Figure 2:**
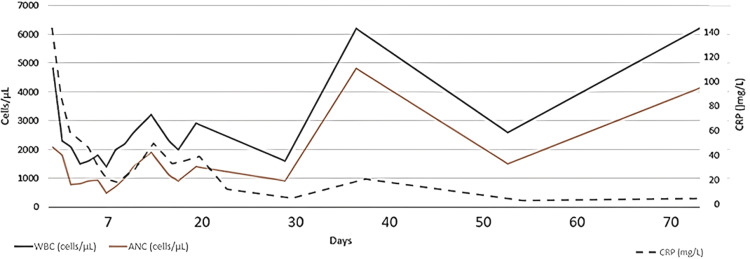
Trends of white blood cell count, absolute neutrophil count, and C-reactive protein

**Figure 3:**
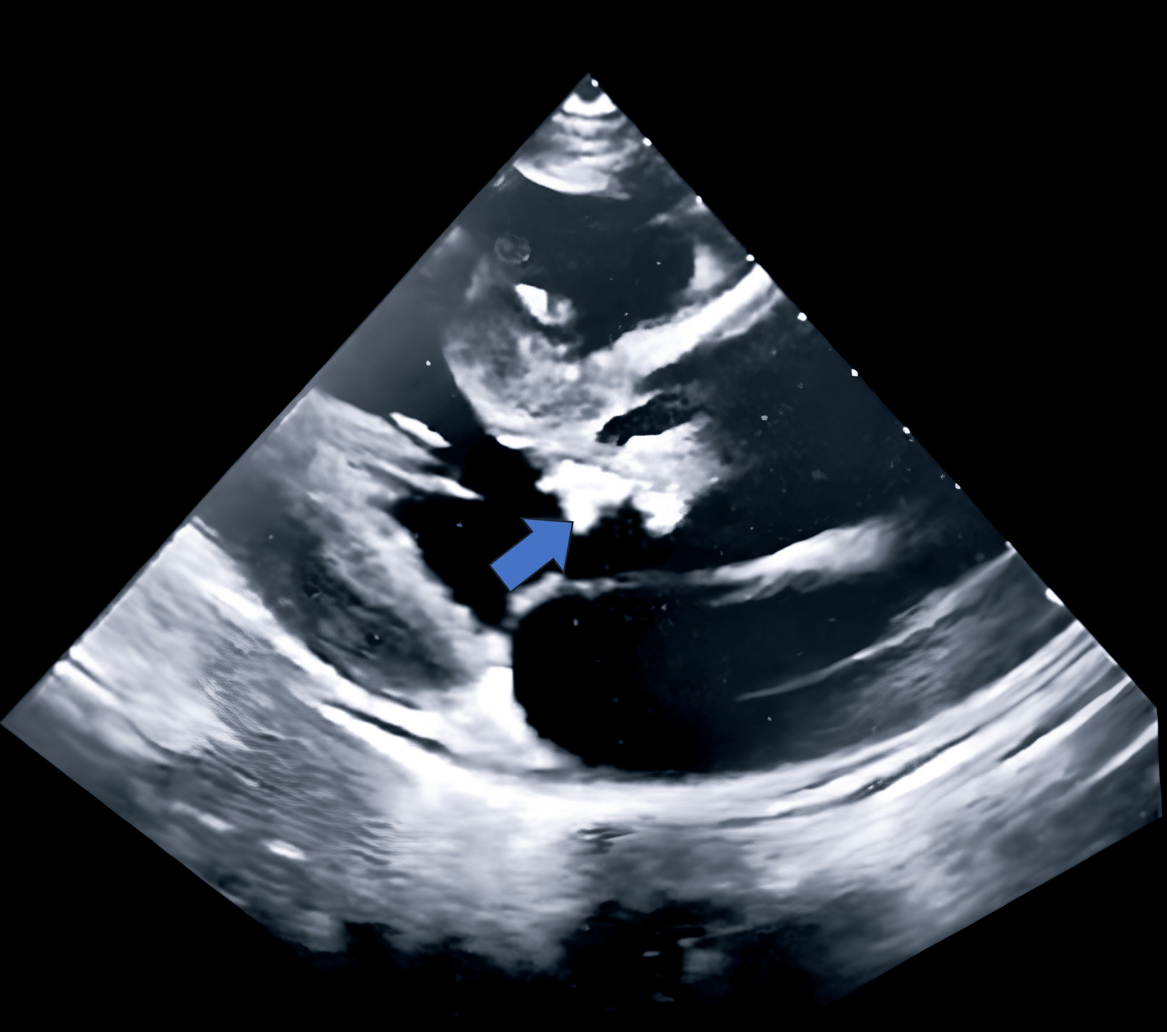
Two-dimensional parasternal long-axis echocardiogram showing large vegetation on the aortic valve (blue arrow)

Laboratory investigations and antibiotic therapy are documented in Table 1, Table 2, Table 3, Figure 1, and Figure 2. Echocardiography revealed vegetations on the aortic valve (Figure 3), fulfilling one of the two major diagnostic criteria for IE. Additional findings included new moderate aortic regurgitation, a mildly dilated left ventricle, but preserved systolic function. IV contrast abdominal CT demonstrated moderate splenomegaly with multiple small lesions suggestive of infarcts.

**Table 1:** Initial laboratory and microbiologic investigations with reference ranges

Test	Result	Reference range
Procalcitonin	1.3 ng/mL	0.02-0.09 ng/mL
C-reactive protein	123 mg/L	0.02-10 mg/L
White blood count	4,600 cells/µL	4,000-11,000 cells/µL
Neutrophils	46%	40-60%
Lymphocytes	35%	20-40%
Monocytes	16%	2-8%
Eosinophils	2%	0-5%
Hemoglobin	9.3 g/dL	11-15 g/dL
Mean corpuscular volume	73 fL	80-100 fL
Platelets	105 ×10⁹/L	150-450 ×10⁹/L
B-type natriuretic peptide	1,494 pg/mL	<100 pg/mL
D-dimer	3.54 mg/L	<0.5 mg/L
Troponin	0.32 ng/mL	<0.04 ng/mL
Respiratory viral panel PCR	Negative for rhinovirus, metapneumovirus, adenovirus, parainfluenza 1-4, RSV, influenza A and B, and SARS-CoV-2	Not applicable
Nasal methicillin-resistant *Staphylococcus aureus* culture	Negative	Not applicable
Blood cultures	Negative on eight aerobic and four anaerobic bottles; one aerobic bottle grew coagulase-negative *Staphylococcus *at 27 hours (deemed contaminant)	Not applicable
Brucella IgG	Negative	<1:160
Q fever (IgG/IgM)	Negative	<1:100
Babesia IgG	Negative	<1:64
Anaplasma IgG	Negative	<1:64
Ehrlichia IgG	Negative	<1:64
Lyme screen	Positive	Not applicable
Lyme IgG/IgM	Negative	Not applicable
Plasma microbial cell-free DNA (Karius)	*Bartonella henselae* 15,842 DNA molecules/µL	<10 DNA molecules/µL

**Table 2:** Bartonella henselae IgG and IgM titers (Lab 1)

Week	IgG titer	Reference range	IgM titer	Reference range
1	>1:2560	<1:320	Negative	<1:100
3	>1:2560	<1:320	Negative	<1:100
9	1:2560	<1:320	Negative	<1:100

**Table 3:** Bartonella henselae IgG and IgM titers (Lab 2)

Week	IgG titer	Reference range	IgM titer	Reference range
13	1:256	<1:64	Negative	<1:100
16	1:512	<1:64	Negative	<1:100
31	1:512	<1:64	Negative	<1:100
36	1:256	<1:64	Negative	<1:100

The second major diagnostic criterion for IE is microbiologic evidence, either a positive blood culture or PCR. Plasma mcf-DNA testing (Karius) was sent after multiple negative blood cultures and returned positive for *B. henselae *DNA, correlating with significantly elevated antibody titers to *B. henselae*. With positive findings on both imaging and mcf-DNA testing, he fulfilled the two major criteria for IE according to the 2023 Duke-ISCVID criteria [6].

Differential diagnosis and treatment

At presentation, the patient was started empirically on ceftriaxone, vancomycin, acyclovir, and doxycycline in view of acute stroke with fever, living in a Lyme-endemic area. Once herpes encephalitis and the likelihood of methicillin-resistant *Staphylococcus aureus* infection were ruled out, acyclovir and vancomycin were discontinued within 24 hours. He continued on ceftriaxone and doxycycline until day 6, when he developed neutropenia. Ceftriaxone was discontinued due to concerns of beta-lactam-associated neutropenia, and rifampin was added. He tolerated the combination of doxycycline and rifampin well.

After an initial decrease, his C-reactive protein (CRP) rose, raising concerns for therapy failure, as both doxycycline and rifampin are primarily bacteriostatic. On hospital day 9, rifampin was replaced with low-dose gentamicin at 3 mg/kg/day. Despite this adjustment, CRP continued to increase, prompting escalation of gentamicin to 7.5 mg/kg/day and addition of ceftazidime at 100 mg/kg/day on day 11.

Outcome and follow-up

Following the increase in gentamicin dose and addition of ceftazidime, CRP began to decline, accompanied by progressive improvement in neurologic deficits and stabilization of the aortic valve vegetation. Gentamicin was discontinued on day 16, completing two weeks of IV bactericidal therapy. He was discharged on hospital day 21 on oral doxycycline and IV ceftazidime via peripherally inserted central catheter.

At home, he developed emesis with doxycycline, prompting a switch to daily azithromycin for a long-term course of four to five months to treat endocarditis. Ceftazidime was stopped on treatment day 30 after he developed a second episode of neutropenia (nadir 500 cells/mm³). He received filgrastim (granulocyte colony-stimulating factor), resulting in recovery of his neutrophil count. Daily azithromycin was reduced to thrice weekly due to persistent hiccups and abdominal pain, which he tolerated well.

Two weeks before completing therapy, imaging revealed significant aortic regurgitation, prompting urgent aortic valve replacement with a 25 mm St. Jude prosthetic valve. Azithromycin was continued for two additional weeks post-surgery, totaling 4.5 months of therapy. Six months post-operation, he remained stable and had regained his baseline weight.

## Discussion

This patient’s case is particularly notable as he developed systemic bartonellosis with endocarditis in the setting of CHD, presenting with an ischemic stroke. IE is less common in children than in adults, with an incidence of 0.43 to 0.69 per 100,000 in children [2]. The primary predisposing factors for IE in children include CHD, RHD, and bacteremia resulting from hospital-acquired infections associated with intravascular devices such as central venous catheters and pacing leads [2,10]. This patient had an elevated risk for IE due to his aortic valvular defects [11]. Approximately 50-70% of pediatric IE cases occur in patients with CHD [11]. This contrasts with adult patients, in whom the most common predisposing heart conditions include degenerative valve disease or prosthetic valve implantation [11].

There is increasing evidence that IE is a significant risk factor for stroke, as seen in this patient [11,12]. In the setting of endocarditis with acute stroke, efforts should focus on minimizing the risk of subsequent cerebral infarcts and, if possible, addressing the underlying pathology of the embolic source. The risk of embolism in endocarditis has been reported to be as high as 10-50%. Specific factors have been identified that elevate the risk of acute ischemic stroke in IE [12,14]. These include a previous history of stroke, infection caused by *Staphylococcus *species, presence of vegetations on the mitral valve, particularly on the anterior leaflet, multivalvular involvement, valvular abscess formation, and vegetations measuring greater than 10 mm in length [12,14].

Valenzuela et al. reported that patients with vegetations larger than 10 mm had a 57% risk of embolic events, compared to 22% for those with vegetations smaller than 10 mm [14]. Additionally, mobile vegetations carried a 48% risk of embolism, compared to 9% for fixed vegetations [14]. These findings are closely linked to vegetation size, as larger vegetation is more likely to be mobile [14]. Consequently, both larger and more mobile vegetations significantly increase the risk of arterial ischemic stroke in patients with IE due to their higher propensity to fragment and embolize [13,14].

BCNIE posed a significant challenge in this case, as long-term mortality has been reported to be higher than in culture-positive endocarditis patients. Plasma mcf-DNA testing has been shown to identify causative pathogens in bacterial bloodstream infections earlier and for a significantly longer duration than conventional blood cultures [6,9]. Although the Karius test demonstrates a clinical sensitivity of 93.7% across various pathogens, its clinical specificity is lower, at 40%. The likelihood of a correct result in this patient was increased, given his exposure to kittens and the fact that* B. henselae* is a common cause of BCNIE [15-17].

Systemic bartonellosis is difficult to treat. Our patient did not respond adequately to the recommended first-line options: doxycycline and rifampin. In vitro studies by Rolain et al. suggest that gentamicin is bactericidal against *B. henselae*, whereas ceftriaxone, a conventional bactericidal antibiotic, is bacteriostatic against *B. henselae *[18]. Studies in adults with *Bartonella *endocarditis have shown improvement with no relapse or persistent disease in patients undergoing early surgical management [18]. Similar reports in the pediatric population are limited, where additional concerns include an increased likelihood of future valve replacements. Vegetations in *Bartonella *endocarditis have been found to be more fibrotic, calcific, and less vascularized, causing structural valve damage even after a reduction in size, which may support early surgical intervention [19,20].

## Conclusions

The diagnosis and management of blood culture-negative IE remain challenging and are often associated with significant morbidity. mcf-DNA testing has proven valuable for achieving an early and accurate microbiologic diagnosis, thereby guiding appropriate antimicrobial therapy. Treatment typically requires a prolonged course of multiple antibiotics, which may predispose patients to antibiotic-related adverse effects. Vegetation caused by B. henselae can compromise the structural integrity of the aortic valve, potentially resulting in aortic regurgitation severe enough to require valve replacement.

## Appendices

Patient’s perspective

The patient reported feeling generally unwell and more symptomatic on the days he had to take doxycycline. At times, he refused his medication, and he found the staff helpful in providing strategies to improve medication compliance. Overall, he stated that his primary motivation for maintaining adherence was to return to school and resume his hobbies.

## References

[1] Yakut K, Ecevit Z, Tokel NK, Varan B, Ozkan M (2021). Infective endocarditis in childhood: a single-center experience of 18 years. Braz J Cardiovasc Surg.

[2] Vicent L, Luna R, Martínez-Sellés M (2022). Pediatric infective endocarditis: a literature review. J Clin Med.

[3] Charles K, Abraham A, Bassi R, Elsadek R, Cockey G (2023). A rare case of Bartonella henselae infective endocarditis causing an embolic cerebrovascular accident. Cureus.

[4] Otto CM, Nishimura RA, Bonow RO (2021). 2020 ACC/AHA guideline for the management of patients with valvular heart disease: executive summary: a report of the American College of Cardiology/American Heart Association Joint Committee on Clinical Practice Guidelines. Circulation.

[5] (2023). Correction to: The 2023 Duke-International Society for Cardiovascular Infectious Diseases criteria for infective endocarditis: updating the Modified Duke Criteria. Clin Infect Dis.

[6] Lin KP, Yeh TK, Chuang YC, Wang LA, Fu YC, Liu PY (2023). Blood culture negative endocarditis: a review of laboratory diagnostic approaches. Int J Gen Med.

[7] Burban A, Słupik D, Reda A, Szczerba E, Grabowski M, Kołodzińska A (2024). Novel diagnostic methods for infective endocarditis. Int J Mol Sci.

[8] Wegdam-Blans MC, Wielders CC, Meekelenkamp J (2012). Evaluation of commonly used serological tests for detection of Coxiella burnetii antibodies in well-defined acute and follow-up sera. Clin Vaccine Immunol.

[9] Eichenberger EM, Degner N, Scott ER (2023). Microbial cell-free DNA identifies the causative pathogen in infective endocarditis and remains detectable longer than conventional blood culture in patients with prior antibiotic therapy. Clin Infect Dis.

[10] Farrar J, Garcia P, Hotez P, Junghanss T, Kang G, Lalloo D, White N (2023). Manson’s Tropical Diseases. Sciences.

[11] Vicent L, Goenaga MA, Muñoz P (2022). Infective endocarditis in children and adolescents: a different profile with clinical implications. Pediatr Res.

[12] Maheshwari R, Wardman D, Cordato DJ (2021). Acute ischaemic stroke in infective endocarditis: pathophysiology and clinical outcomes in patients treated with reperfusion therapy. Immuno.

[13] Nitsch L, Shirvani Samani O, Silaschi M (2023). Infective endocarditis and stroke: when does it bleed? A single center retrospective study. Neurol Res Pract.

[14] Valenzuela I, Hunter MD, Sundheim K (2018). Clinical risk factors for acute ischaemic and haemorrhagic stroke in patients with infective endocarditis. Intern Med J.

[15] Babady NE (2021). Clinical metagenomics for bloodstream infections: is the juice worth the squeeze?. Clin Infect Dis.

[16] Eichenberger EM, de Vries CR, Ruffin F (2022). Microbial cell-free DNA identifies etiology of bloodstream infections, persists longer than conventional blood cultures, and its duration of detection is associated with metastatic infection in patients with Staphylococcus aureus and gram-negative bacteremia. Clin Infect Dis.

[17] Rolain JM, Maurin M, Raoult D (2000). Bactericidal effect of antibiotics on Bartonella and Brucella spp.: clinical implications. J Antimicrob Chemother.

[18] Satake K, Iijima K (2023). Ceftriaxone-induced neutropenia successfully treated with alternative β-lactam antibiotics: a case report and review of the literature. Cureus.

[19] Ordaya EE, Abu Saleh OM, Mahmood M (2023). "Let the cat out of the heart": clinical characteristics of patients presenting with blood culture-negative endocarditis due to Bartonella species. Open Forum Infect Dis.

[20] Lepidi H, Fournier PE, Raoult D (2000). Quantitative analysis of valvular lesions during Bartonella endocarditis. Am J Clin Pathol.

